# Porcelain Inlays

**Published:** 1904-07

**Authors:** H. E. Phillips

**Affiliations:** Canton, O.


### PORCELAIN INLAYS.

By H. E. Phillips, D. D. S., Canton, O.

(Written for *The American Journal of Dental Science.*)

The editor has asked that I "keep in mind that it is for beginners" I write, "giving some practical hints" on porcelain inlay work, seeming to ignore the fact that most of us are of the kindergarten class, only a few having attained anything akin to perfection. However, all along the way between the two stations are numerous toilers striving hard for results, and getting them, too.

But I am going one better and address the man who has not yet begun, though may be thinking seriously of doing so.

The most "practical hint" I can give to a dentist contemplating becoming a beginner is this: Spend a day or several, if possible, under the instruction of a porcelain worker who has acquired knowledge and skill and from him learn your A B C's. Then go to your own laboratory and go over the lesson again and again. In other words, take teeth that have been previously immersed in alcohol and drill cavities in every conceivable position in which you would consider a porcelain inlay practical, make a matrix and inlay for every one of them, trying every shade in your outfit.

Be as scrupulously particular as though the tooth was in the mouth of one of your best patients. Don't think of experimenting on your patients. It is not necessary as you can do all that in your laboratory with porcelain.

Take in all the clinics on the subject within your reach. No matter who the demonstrator, you can learn something worth while.

Following this course you can know more in a month than you could possibly learn in a year should you try to "pick it up" alone. Let me picture to you a single incident that, with a few variations, will represent the experience of a vast number of "beginners": Dr. X., of whom I speak, is more than ordinarily skillful as an operator and successful as a practitioner. After absorbing such information as he could get from the various dental journals he purchased an expensive furnace and all the other articles necessary and conducive to success and then informed me there would be "something doing" at his office in about forty-eight hours.

Result: After one year of misdirected effort, during which time he has been unable to set a single inlay that would satisfy a blind man, the outfit has been recently relegated to a top shelf in the laboratory and the doctor is disgusted with and does not believe in porcelain. A more patient man would have won out after much effort. Remember, porcelain inlay work requires more patience, skill and delicacy than anything the dentist is called on to do, hence, the fallacy of trying to make and place a creditable inlay without first learning something of the procedure from an expert. The preparation of the cavity is a phase of the subject that as a rule does not receive as much attention from the novice as it should. He is so anxious to get at the building of the inlay that all else is passed over. When he has met with a few failures in consequence he then begins to look to the foundation of his work. I cannot be too emphatic in my reference to shaping of the cavity, because it is really the most important factor conducive to success. You hear and read much of the color and shadow question and the making of the matrix, but as you progress with your laboratory experiments, thereby becoming familiar with the colors at your disposal, these features will not worry you so much when you come to do practical work. But your patience and skill will be taxed to their utmost very often when preparing a cavity, even after you have gotten well on. Again the color, the elimination of shadow and the making of the matrix are all dependent on the proper preparation of the cavity. When the "beginner" can successfully insert a porcelain inlay, he is repaid for all the expense of time and money spent in his effort to properly fit himself, for there is nothing so pleases your patient or so satisfying to the dentist from an aesthetic viewpoint as a well-made porcelain inlay.

---

### SOME INLAY POINTS.

By Alexander Jameson, D. D. S.,
Indianapolis, Ind.

(Written for *The American Journal of Dental Science.*)

